# A novel manometric test to assess inherent biodegradability of complex and non-soluble chemicals

**DOI:** 10.3389/fmicb.2026.1822141

**Published:** 2026-04-24

**Authors:** Philippe Marchal, Alicia Lessentier, Maël Guedj, Anaelle Torres, Valérie Jomain, Neil Wang, Sophie Galinat Delpech, Emie Lacombe, Céline Guillaume, Min Yan Wu, Yi Gong, Christophe Moineau, Céline Coste, Feng Wang, D. James Wilson, Mickael Cregut

**Affiliations:** 1 SYENSQO Research Innovation Center of Lyon, Biomattech Platform, Saint-Fons, France; 2 SYENSQO Industrial Function, TERA, SILEX-2, Lyon, France; 3 SYENSQO Industrial Function, TERA, Aubervilliers, France; 4 Laboratoire du Futur, (LOF)─SYENSQO─CNRS─Université de Bordeaux, UMR 5258, Pessac, France; 5 SYENSQO China, Xinzhuang Indus, Shanghai, China; 6 SYENSQO Industrial Function, Performance and Care, Lyon, France; 7 SYENSQO Research Innovation Center of Paris, Biomattech Platform, Aubervilliers, France

**Keywords:** biodegradability, inherent biodegradability, manometric respirometry, non-soluble chemical biodegradation, OECD 301F, OECD 302B, polymer biodegradation

## Abstract

This study presents a modified manometric test for inherent biodegradability designed to assess the inherent biodegradability of complex and poorly soluble chemicals, including polymers with adsorption behavior. The method is derived from OECD 301F and adjusted toward inherent conditions, with higher biomass loading and an optimized Mass/ Food ratio. It addresses limitations of traditional tests, particularly for polymers and substances with low solubility or adsorption properties, while offering enhanced automation. The study used chemicals such as sodium benzoate and polyhydroxybutyrate to assess the modified manometric test for inherent biodegradability using OxiTop®-IDS pressure sensors, comparing it against standard OECD 301F (manometric pressure) and OECD 302B (DOC removal). The modified manometric test for inherent biodegradability enabled the assessment of biodegradability for pure chemicals and of overall biodegradability for complex polymers. Reference soluble chemicals that were evaluated achieved biodegradation levels between 78 and 100%. For polymers, the method overcame solubility and adsorption challenges, with biodegradation levels ranging from 76 to 100% for biodegradable polymers. Notably, in the BMT-C* polymer case study, OECD 302B indicated high apparent biodegradation based on DOC removal, whereas the modified manometric test for inherent biodegradability showed substantially lower biodegradation, highlighting the potential of DOC-based methods to overestimate biodegradation in such systems. These results support the potential of the method as an interesting option for determining overall inherent biodegradability of complex polymers. Significance: The modified manometric test for inherent biodegradability extends inherent biodegradability testing to low-solubility and adsorptive substances while maintaining agreement of 301F/302B results for soluble references (78–100% in 28 d).

## 1 Introduction

Over the last few decades, there has been a growing societal demand for more sustainable and eco-friendly chemicals with improved environmental profiles. The European Commission published a Chemical Sustainability Strategy aiming to achieve a toxic-free environment [EC (European Commission), 2020]. This strategy includes strong regulatory incentives for industry to develop substances that would degrade rapidly if released into the environment. The European Commission thus developed recommendations for a Safe and Sustainable by Design approach, in which the assessment of biodegradation potential is a key criterion for the development of new products [EC (European Commission), 2022]. As an example, the REACH regulation allows for three successive tiers of testing: initial screening for ready biodegradability (OECD 301 series), followed by inherent ultimate biodegradability screening (OECD 302 series), and, if necessary, simulation tests (OECD 307–309) [ECHA (European Chemicals Agency), 2023]. However, OECD 307–309 are very expensive and time-consuming making them unsuitable for routine use in new product development where cheap and faster screening tools are largely preferred.

Ready and inherent biodegradability tests check for the rapid and full mineralization of the test substance over time when incubated under aerobic conditions with an inoculum of either natural origin (e.g., river water) or sourced from the activated sludge of a municipal wastewater treatment plant. In both tests, the substance is added as the sole source of organic carbon (i.e., the sole source of energy). The main differences are the biomass/organic carbon ratio and the quantity of biomass introduced in the incubation system, which are both higher in the inherent than in the ready biodegradability tests. This makes ready biodegradability testing more stringent because of the reduced likelihood to introduce at least one consortium of microorganisms able to use the substance as a source of energy. A substance which meets the ready biodegradability criteria is considered to mineralize rapidly in most natural environments (see OECD TG 301 for more information on the criteria). A substance which does not meet these criteria may however still mineralize rapidly in the natural environment. This may be further investigated in the inherent biodegradability test (OECD 302 series) which checks more exhaustively for the existence of microorganism consortia able to feed on the test substance. There is however less scientific consensus on how the results of the test can be extrapolated in terms of mineralization kinetics in the natural environment. While these screening methodologies can effectively assess the biodegradability of easy-to-test mono-constituent substances, challenges remain for polymers and poorly water-soluble molecules (< 100 mg/L), mostly due to their low bioavailability for microorganisms. The scientific literature proposes several adaptations, known as “bioavailability improvement methods” (BIM), which can address many of the physico-chemical properties that limit bioavailability (such as adsorption to glass and/or suspended solids, poor water solubility, and ready partitioning to air) (Sweetlove et al., 2016). These methods have been shown to improve the reliability of certain OECD TG 301 tests, such as OECD 301B, 301D and 301F, but are not applicable to all tests in the series. However, the OECD TG 302 series is limited to substances which are both highly water-soluble (> 1 g/L) and non-sorptive. Thus, there is a gap in the screening test arsenal for the other classes of substances, particularly poorly water-soluble, sorptive, or compositionally complex chemicals, including polymers and other multi-constituent materials, which are often among those that fail OECD TG 301 pass criteria. As discussed in the literature, under ready biodegradability test conditions, these properties may limit bioavailability and interfere with the expression and measurement of biodegradation (Strotmann et al., 2023). This has led to the need to adapt standard methods or develop alternative protocols to better evaluate the biodegradation potential of complex or poorly soluble chemicals (Pagga, 1997; Pires et al., 2022; Menzies et al., 2023; Strotmann et al., 2023).

In the case of polymers, biodegradation generally proceeds through depolymerization followed by assimilation and mineralization, where microbial activity converts the material into CO2 and/or CH4 depending on the aeration conditions (Cregut et al., 2013). This process involves two main phases: fragmentation by extracellular enzymes and subsequent assimilation of the resulting oligomers or monomers, as described by Priya et al. (2022) and Amobonye et al. (2021). In parallel with standardized ready and inherent biodegradability tests, several studies have investigated non-guideline miniaturized and high-throughput screening formats which, although not directly applicable for regulatory submissions under REACH, can help laboratories cope with the growing demand for screening tests driven by such regulations (Cregut et al., 2014; Ott et al., 2020). Pires et al. (2022) also highlighted the importance of combining multiple tests to capture a broader range of environmentally relevant conditions and support a more comprehensive evaluation for each polymer type. From a complementary perspective, Geerts et al. (2021) highlighted that, for multi-constituent substances, summed parameters alone may not be sufficient to conclude on the non-persistence of all individual constituents. In such cases, complementary approaches based on catabolic grouping and read-across can support a more constituent-level interpretation of biodegradability outcomes. In the same perspective, integrative readouts based on carbon balance, such as the Ultimately Transformed Organic Carbon (UTOC) concept, have been proposed to better describe the partitioning of carbon between mineralization, biomass, and residual fractions in complex or poorly soluble systems (Brillet et al., 2018). However, such integrative approaches remain insufficient to fully unify biodegradability assessment across all physicochemical configurations, and their applicability may also be limited for sorptive chemicals, for which sorption onto biomass can bias the interpretation of transformed versus truly mineralized carbon (Pagga, 1997; Strotmann et al., 2023).

Historically, the scientific community has explored various strategies to improve the biodegradability assessment of polymers, including the addition/use of depolymerizing agents or chemical modifications to enhance solubility (Gupta and Deshmukh, 1982a, 1982b; Piemonte et al., 2013; Li et al., 2024). However, these methods were largely abandoned due to their high cost and complexity, which hindered reliable biodegradation assessment. From a regulatory perspective, as seen in frameworks like REACH, the Environmental Protection Agency (EPA), and the Ministry of International Trade and Industry (MITI), biodegradation assessment mainly relies on simple screening tests that use the test substance as the sole carbon source. The stringent nature of these tests ensures that the biodegradability of a substance meets the energy, redox, and nutrient requirements of the environmental inoculum, potentially supporting cell growth.

This article proposes a significant improvement in assessing the inherent biodegradation of complex organic substances. To date, OECD 302C remains the only recognized method for testing the inherent biodegradability of insoluble polymer particles in the context of the REACH restriction on microplastics (Regulation (EU) 2023/2055). Specifically, it requires the preparation of several inocula, which must be collected from various environments and maintained for 3 to 6 months before use. In addition, the method relies on a BOD-meter such as those based on coulometry as specified according to the annex specifications. However, as highlighted by Kayashima et al. (2014) for OECD 301C, the inoculum preparation strategy inherited from the original MITI methods can constrain biodegradation activity and diminish test performance when compared with approaches using fresh environmental inocula. Because OECD 302C is built on the same conceptual framework, similar limitations can reasonably be anticipated. Moreover, using inocula from cultivated sludges coming from various environments and notably industrial sewage plants can lead to an overestimation of the environment’s capacity to host degrading microorganisms, as these sources may not accurately represent natural microbial communities. Studies have shown that the microbial composition of activated sludge can vary significantly across sources and over time, affecting both test reproducibility and the interpretation of biodegradation outcomes (Vázquez-Rodríguez et al., 2011). Similarly, Mezzanotte et al. (2005) demonstrated that different inocula yielded divergent biodegradation results, and that acclimation did not necessarily improve reliability. Together, these findings indicate that the inocula required by standardized methods such as OECD 302C may be poorly aligned with real environmental conditions, thereby reducing their predictive value. In parallel, although OECD 302B is more widely used for inherent biodegradability screening of soluble substances, its applicability may also be limited for sorptive or polymeric chemicals. In such cases, difficulties in solubilization, centrifugation and filtration, together with sorption onto sludge or glassware, may bias DOC-based measurements and lead to an apparent removal of organic carbon that cannot be directly interpreted as true biodegradation (Pagga, 1997). Although standard biodegradation tests do not faithfully reproduce real environmental conditions, considering the probability of biodegradation as proposed by Thouand et al. (2011) and further developed by François et al. (2016) provides a better link between laboratory results and the potential of natural environments to host microorganisms capable of ensuring degradation.

**Figure 1:**
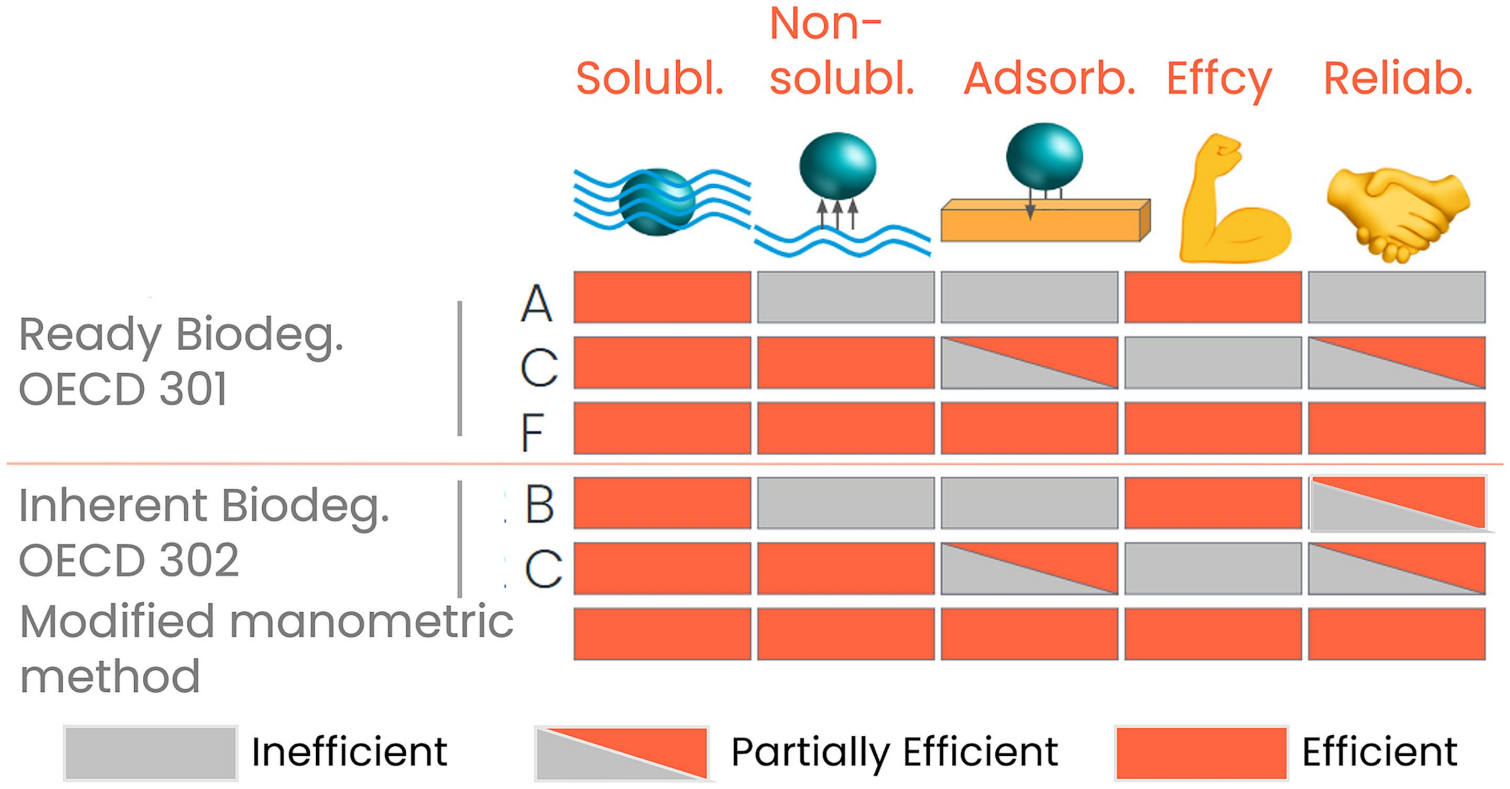
Advantages of the development of a manometric measurement to assess inherent biodegradability compared to the existing methods: with “Solub.” for soluble chemicals; “Non-Solub.” for chemicals with a relative low-solubility generally lower than 1 g L^−1^ presenting some challenges in OECD 30X tests; “Adsorb.” for chemicals susceptible to adsorption; “Effcy” relative to the potential power of the test by using a relatively large and fresh inoculum; “Reliab.” here refers to the conventional acceptance of the test by the scientific community. However, data from OECD 301/2C tests are often considered disconnected from real environmental conditions because the procedures involve mixing and cultivating the inoculum. This process introduces stochastic variability, allowing microorganisms to evolve in unpredictable ways, which does not reflect natural environments and OECD 301A/302B present certain limitations due to the potential difference of solubility of the biodegradation products whereas OECD 302B is generally accepted to clarify the inherent potential.

To address these limitations, we have developed a modified methodology based on an adaptation of the manometric OECD 301F test, specifically tailored for evaluating poorly water-soluble mono-constituent substances and those that present a significant risk of adsorption, including polymers. Our approach aims to optimize test conditions to closely mimic the OECD TG 302B, thereby increasing the likelihood of accurately assessing biodegradation. This method not only measures biodegradability but also enhances the ability to assess the inherent degradation potential of complex substances, supporting the development of an alternative manometric approach for inherent biodegradability assessment (Figure 1). A comparative analysis of this method against existing standards is also provided.

## 2 Materials and Methods

### 2.1 Chemicals used in the study

Several chemicals of analytical grade were used in this study (Table 1): Sodium Benzoate (CAS: 532–32-1), ref.: 27694.293-1KG, VWR, France; Diethylene Glycol (CAS: 111–46-6), ref.: 32160-500 mL, Sigma Aldrich, France; Inulin from Chicory (CAS: 9005-80-5), ref.: I2255-10G, Sigma Aldrich, France; Jaguar S (CAS: 9000-30-0), ref.: V21073529-100G; SYENSQO, USA; Starch (CAS: 9005-25-8), ref.: S5296-5G, Sigma Aldrich, France; Polyhydroxybutyrate (CAS: 26063–00-3), ref.: 403105-10G, Sigma Aldrich, France; Sodium 5-Sulphoisophthalate (SSIA) (CAS: 6362-79-4), ref.: 6222026000, Future Fuel, USA; Polymer BMT-C* (internal prototype polymer composed of 4 monomers including 15% SSIA), ref.: no-ref, SYENSQO, India.

**Table 1:** Substances of reference used in the study to validate the functionality of the modified manometric test for inherent biodegradability.

Reference chemicals	CAS	Pure chemical or Polymer	Solubility > 1 g L^−1^	Observed or expected sorption	Expected compatibility with OECD methods	Methods applied in the study	Status for OECD 302B
Sodium Benzoate	532-32-1	Pure Chemical	Soluble	Not identified	301F and 302B	301F, 302B, modified inherent manometric test	Tested
Diethylene Glycol	111-46-6	Pure chemical	Soluble	Not identified	301F and 302B	301F, 302B, modified inherent manometric test	Tested
Monomer #1	Confidential	Pure chemical	soluble	Not identified	301F and 302B	301F	Not tested
Monomer #2	Confidential	Pure chemical	Soluble	Not identified	301F and 302B	301F	Not tested
Monomer #3	Confidential	Pure chemical	Soluble	Not identified	301F and 302B	301F	Not tested
Sodium 5-Sulphoisophthalate (SSIA)	6362-79-4	Pure chemical	Soluble	Not identified	301F and 302B	301F, 302B, modified inherent manometric test	Tested
Inulin	9005-80-5	Polymer	Soluble	Unknown	301F and 302B	301F, 302B, modified inherent manometric test	Tested
Jaguar S	9000-30-0	Polymer	Soluble	Unknown	301F and unknown	301F, attempted 302B, modified inherent manometric test	Started then discontinued
Starch	9005-25-8	Polymer	Poorly soluble	Suspected	301F and unknown	301F, attempted 302B, modified inherent manometric test	Started then discontinued
Polyhydroxy-butyrate	26063-00-3	Polymer	Insoluble	Unknown	301F	301F, modified inherent manometric test	Not tested
Polymer BMT-C*	Monomer #1 + #2 + #3 = 85% w/w Monomer #SSIA = 15% w/w	Polymer	Soluble	Unknown	301F and unknown	301F, attempted 302B, modified inherent manometric test	Tested (initially considered compatible)

### 2.2 Inoculum used in the study

The inoculum employed in this experiment comprised washed activated sludge sourced from the Auvergne Rhône Alpes region, France. The sludge was sampled and aerated under overnight agitation to decrease organic carbon content and consequently reduce endogenous respiration, as recommended by the OECD 301 guideline.

Following the overnight incubation, a final procedure was conducted to eliminate excess organic carbon. This involved three successive centrifugation steps (6,000 RCF for 15 min, 15 °C) to remove the liquid content. Biomass was quantified as MLSS (g·L^−1^) (Mixed Liquor Suspended Solids) following gravimetric drying at 105 °C to constant mass. MLSS was chosen rather than MLVSS (Mixed Liquor Volatile Suspended Solids) to maintain coherence with Zahn-Wellens practice; MLSS values and the Mass to Food ratio (M/F ratio: mg MLSS per mg DOC) are reported for the different runs. Inoculum stock solutions were resuspended at 5 g L^−1^ MLSS in the mineral medium specified for the biodegradation assay.

### 2.3 Composition of the medium

The composition of the medium of the biodegradation assay was 85 mg L^−1^ KH_2_PO_4_, 217.5 mg L^−1^ K_2_HPO_4_, 334 mg L^−1^ Na_2_HPO_4_, 27.5 mg L^−1^ CaCl_2_, 11.5 mg L^−1^ MgSO_4_ and 0.100 mg L^−1^ FeCl_3_. The pH of the medium was adjusted to 7.2 by the addition of a 1 M aqueous solution of HCl.

### 2.4 Ready biodegradation tests

The ready biodegradability of the “test item” was assessed using the standardized manometric OECD 301F test (OECD, 1992a, 1992b). This assay utilized the OxiTop®-IDS sensor available from Xylem Analytics, France. The biodegradation assay proceeded by incubating the washed inoculum at a final concentration of 30 mg MLSS L^−1^ in the presence of the “test item.” This condition was designated as the biodegradation assay. Biodegradation was monitored in BOD flasks incubated at 22 °C ± 2 °C using TS608/4i incubation chambers from Xylem Analytics, France. Biodegradation measurements were based on daily recorded variations in manometric pressure, using the OxiTop®-IDS sensors. Biodegradability was then quantified as a percentage relative to the amount of oxygen required to mineralize the “test item.”

Additional testing conditions incorporated a reference substance, such as starch, used in the assay to evaluate both the functionality of the inoculum and the non-toxicity or low toxicity of the “test item.” To confirm the functionality of the inoculum, the biodegradability of starch was assessed at an equal amount of substance, expressed in mgO_2_ according to the Chemical Oxygen Demand (COD) or Theoretical Oxygen Demand (ThOD). This setup was referred to as the reference control. Lastly, to confirm the non-toxicity of the “test item,” both starch and the test item were combined at the initial concentrations in an assay, and the biodegradability of the mixture was evaluated following the OECD 301 guidelines. This condition was named the toxicity control.

### 2.5 Inherent biodegradation tests

#### 2.5.1 Standard OECD 302 B

The inherent biodegradability of the “test item” was assessed using the measurement of the disappearance of dissolved organic carbon, following the OECD 302B guideline (OECD, 1992a, 1992b). This assay utilized large vessels commonly filled with 1 L of mineral medium, in which successive samples of mineral medium were analyzed using a COTmeter or via a COD measurement to assess the remaining Dissolved Organic Carbon (DOC). Measurements of DOC were processed after a centrifugation step at 6,400 RCF, during 15 min. The DOC remaining in the supernatant was filtered through a 0.45 μm mesh and measured (*i*) via a COTmeter with the use of a subtractive approach measuring both Total Carbon and the Inorganic Carbon following the instructions of the manufacturer (model: TOC-L CHvNS; COTmeter Shimadzu, Fr); (*ii*) or via a COD measurement using a DR-3900 Spectrophotometer with the use of Hach test kits (LCK 114 & LCK 314), following the manufacturer’s instructions (Hach, Fr).

The biodegradation assay then proceeded by incubating the washed inoculum at a final concentration ranging from 100 mg to 500 mg MLSS L^−1^ in the presence of the “test item” for which the nominal concentration ranged from 50 mgCOD L^−1^ to 500 mgCOD L^−1^. This condition was designated as the biodegradation assay. Biodegradation was incubated at 25 °C ± 0.5 °C using an incubation chamber Multitron (Infors, Fr). Biodegradability was then quantified as a percentage of dissolved carbon that disappeared in the assay, following the technical guideline instructions.

OECD 302B was applied to Sodium Benzoate, Diethylene Glycol, Inulin, SSIA, and polymer BMT-C*. Jaguar S and Starch were initially considered for OECD 302B but the approach was discontinued because adsorption and/or separation issues were observed within the first 3 h of testing, making DOC-based interpretation unsuitable. Polyhydroxybutyrate was not investigated with OECD 302B because its particulate/flaked form was considered incompatible with the method.

#### 2.5.2 Modified manometric test for inherent biodegradability

**Figure 2:**
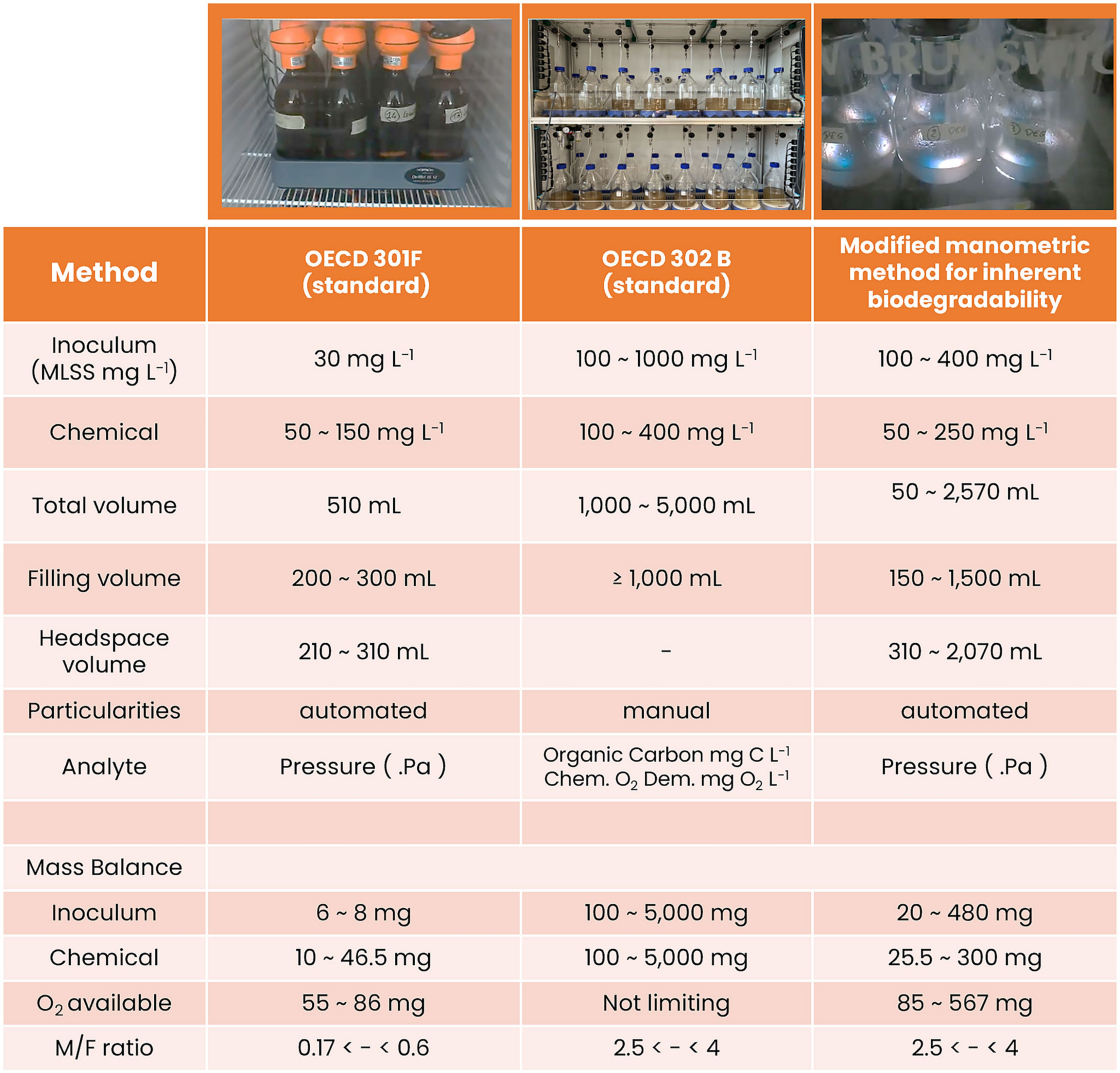
Setup of the different methods used to compare the performance of the modified manometric test for inherent biodegradability compared to the standards proposed by the OECD guidelines.

The modified manometric test for inherent biodegradability was carried out to qualify the inherent biodegradability of the test item. To proceed with this evaluation, the biodegradation assessment is carried out using OxiTop®-IDS sensors enabling manometric assessment of the oxygen consumption as previously done for the OECD 301F test. This assay utilized the OxiTop bottles and jars with total volumes ranging from 510 mL to 2,540 mL, available from Xylem Analytics, France (Figure 2). The biodegradation assay then proceeded by incubating the washed inoculum at a final concentration ranging from 100 to 500 mg MLSS L^−1^ in the presence of the “test item,” in accordance with the OECD302B guidelines. The ratio of food and biomass generally used to characterize inherent biodegradability ranged from 2.5 to 4 as expressed in mg DOC L^−1^ vs. mg MLSS L^−1^. This range was also selected in accordance with the OECD 302B guideline, which recommends maintaining an inoculum-to-substrate ratio between 2.5 and 4 to ensure reliable assessment of inherent biodegradability (OECD, 1992b). This ratio allows maintaining sufficient microbial activity while avoiding substrate overloading or inhibitory effects. However, in addition, it is necessary to verify that sufficient oxygen is available in the headspace to sustain biodegradation throughout the test. If needed, the filling volume of the medium should be adjusted accordingly to prevent oxygen limitation. In practice, the ranges presented in Figure 2 provided appropriate conditions, ensuring both adequate oxygen availability and a low background signal, with endogenous respiration typically around 1 to 1.3 mgO₂ per mg biomass over 28 days and low variability for the sludge used in this study (Supplementary data, Table A1 in Supplementary material).

Biodegradation was monitored in airtight bottles equipped with a manometric sensor and with filling volumes ranging from 150 mL to 1,500 mL according to the quantity of oxygen required for the biodegradation, which corresponded to the different size of bioreactors commercially available from Xylem Analytics. The biodegradation assays were incubated in the dark in incubators from 22 °C to 25 °C +/−0.5 °C using the TS608/4i incubation chambers or a Multitron (respectively Xylem Analytics, Fr; Infors, Fr) depending on flask size. Biodegradation measurements were based on daily recorded variations in manometric pressure, using the OxiTop®-IDS sensors. Biodegradability was then quantified as a percentage relative to the amount of oxygen required to mineralize the “test item.”

Additional testing conditions incorporated a reference substance, such as starch, used in the assay to evaluate both the functionality of the inoculum and the non-toxicity or low toxicity of the “test item.” To confirm the functionality of the inoculum, the biodegradability of starch was assessed at an equal amount of substance, expressed in mgO_2_ according to its COD or ThOD. This setup was referred to as the reference control. Lastly, to confirm the non-toxicity of the “test item,” both starch and the test item were combined at the initial concentrations in an assay, and the biodegradability of the mixture was evaluated following the consumption of oxygen calculation.

### 2.6 Calculation of biodegradability

For the condition in which biodegradation is assessed through the measurement of a pressure sensor, The biodegradation test is carried out with Oxitop IDS sensors (Xylem, Fr), in airtight reactors from Xylem analytics. The apparatus reports pressure in hPa. For calculations, ΔP is converted to Pa (hPa × 100). To ensure that the measured pressure decrease strictly reflects oxygen consumption, carbon dioxide released by microbial respiration was quantitatively trapped using sodium hydroxide (NaOH) pellets placed in a quiver attached to the bottle head and kept above the liquid phase. Under these conditions, CO_2_ is converted to non-volatile carbonate, and the only expected headspace pressure change arises from O₂ uptake. This setup ensures that the variation in manometric pressure exclusively reflects oxygen consumption.

Under these conditions an accurate measure of oxygen consumption is calculated in mg O_2_ (mO_2_), from the measurement of the decrease of the atmospheric pressure, as described in Equation 1, with P as Pressure in Pa, V as headspace gas volume in m^3^, R as a constant in J.mol^−1^. K^−1^, T as Temperature in °K and MW as Molecular Weight of Oxygen in m. M^−1^.

Equation 1 (Conversion of pressure drop to oxygen mass):


$$mO_{2}=\frac{\mathrm{\Delta P}\times V\times \mathrm{MW}}{R\times T}$$ (1)

Thereafter, the residual mass of O_2_ consumed in the flask containing the test item, subtracted from the O_2_ consumption of the blank inoculum, is expressed per reactor liquid volume V_L_ to establish the Biological Oxygen Demand (BOD) (Equation 2).

Equation 2 (Biological Oxygen Demand (BOD))


$$\mathrm{BOD}=\frac{mO_{2,\;\text{test}}-mO_{2,\text{blank}}}{V_{L}}$$ (2)

Biodegradation percentage (BioD) is then calculated by expressing the measured BOD as a percentage of the Theoretical Oxygen Demand (ThOD) (Equation 3). If the elemental composition of the product is not known, its chemical oxygen demand can be used instead of its theoretical oxygen demand.

Equation 3 (Percentage of biodegradation through BOD measurement):


$$\text{BioD}\;(\%)=100\times \frac{\mathrm{BOD}\;}{\text{ThOD}}$$ (3)

For the case in which biodegradation is assessed through the measurement of the disappearance of organic carbon, the decrease in dissolved organic carbon (DOC) is measured to evaluate biodegradability. The residual organic carbon is quantified in a reactor containing the test item. The amount of organic carbon that disappears is determined by subtracting the residual organic carbon from that in a blank reactor and comparing it to the initial amount present at the starting point (Equations 4, 5, depending on the use of the DOC or COD measurement).

Equation 4 (Percentage of biodegradation through DOC measurements):


$$\begin{array}{l} \text{BioD}\;(\%)=\\ \;\;\;\;100\times \frac{\left(\mathrm{DO}C_{t=0}-\mathrm{DO}C_{t})-(\mathrm{DO}C_{\text{blank},t=0}-\mathrm{DO}C_{\text{blank},t}\right)}{\mathrm{DO}C_{t=0}} \end{array}$$ (4)

Or

Equation 5 (Percentage of biodegradation through COD measurements):


$$\begin{array}{l} \text{BioD}\;(\%)=\\ \;\;\;\;100\times \frac{\left(\mathrm{CO}D_{t=0}-\mathrm{CO}D_{t})-(\mathrm{CO}D_{\text{blank},t=0}-\mathrm{CO}D_{\text{blank},t}\right)}{\mathrm{CO}D_{t=0}} \end{array}$$ (5)

For pure chemicals and for polyhydroxybutyrate, whose composition and purity were known, biodegradation percentages were calculated using the theoretical oxygen demand (ThOD), derived from the molecular formula according to OECD 301 (OECD, 1992a, 1992b), with adaptation to the appropriate nitrogen oxidation state (ThOD_NH4_ or ThOD_NO3_, when relevant). For polymeric substances subject to compositional variability and for which no unambiguous molecular formula could be assigned, biodegradation percentages were instead expressed relative to the measured chemical oxygen demand (COD).

### 2.7 Quality assurance/acceptance criteria

(*i*) Reference control (starch) ≥ 60% biodegradation in ≤ 14 days, to validate the biotic reference; (*ii*) Inoculum blanks oxygen consumption must be ≤ 60 mg O_2_ L^−1^ over 28 days, confirming that background organic contamination remains limited; this is a mandatory validity criterion for the OECD 301F assay. For the modified manometric test for inherent biodegradability, this threshold was adapted to account for the higher biomass loading, with a limit set at ≤ 150 mg O_2_ L^−1^; (*iii*) Replicate RSD ≤ 20%; (*iv*) pH endpoint 6.0–8.5; (*v*) Leak test and Na_2_SO_3_ positive-control confirm instrument response.

Results are expressed as mean values ± standard deviation based on replicate measurements. At this stage, these replicate data are intended to indicate internal experimental variability only; the limited number of tested substances and conditions does not allow a comprehensive statistical evaluation of method robustness, reproducibility, or inter-method comparability.

## 3 Results and discussion

### 3.1 Evaluation of the modified manometric test for inherent biodegradability using reference substances

**Figure 3:**
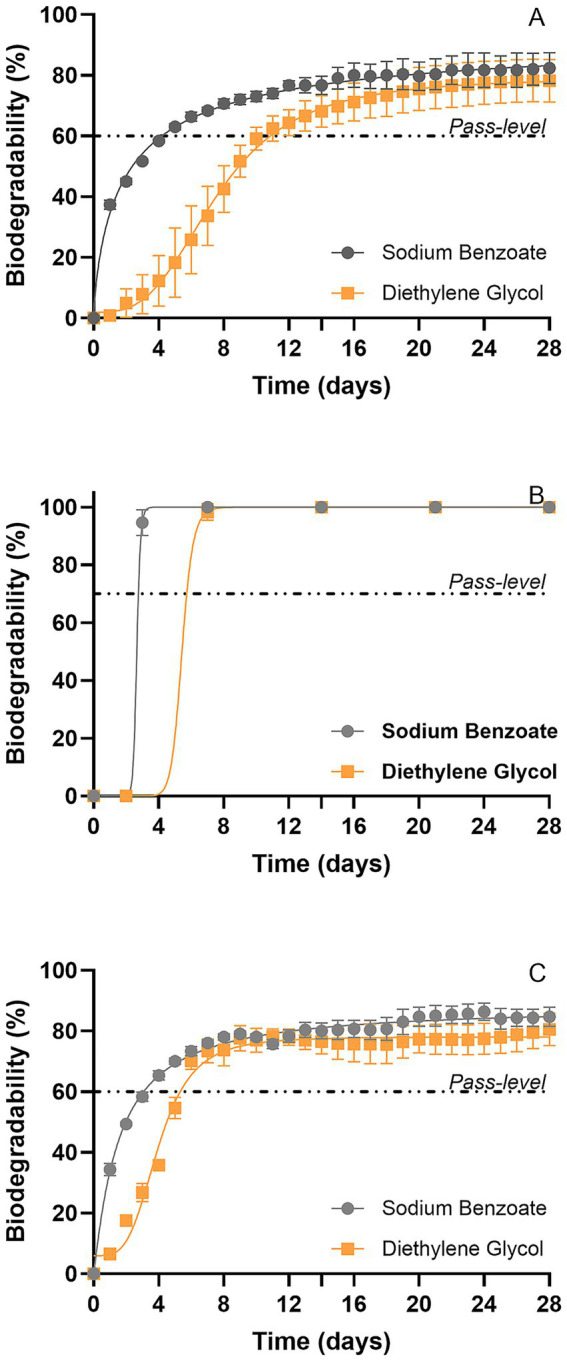
Biodegradability using OECD 301F **(A)**, OECD 302B **(B)**, and the proposed modified manometric test for inherent biodegradability **(C)** with pure substances considered as reference chemicals. For manometric measurements, biodegradation percentages were calculated relative to ThOD.

The biodegradability of the substances was determined using both the standardized manometric assay OECD 301F test and the modified manometric test for inherent biodegradability, using OxiTop®-IDS sensors available from Xylem Analytics (France) and when possible, the standard OECD 302B test. Applying the different biodegradation tests on the reference chemicals allowed us to assess the potential of biodegradation of these substances with an urban sludge sampled from the Auvergne Rhône Alpes region (France) and to investigate the adequation of the test methods to reveal the biodegradation potential of the different compounds according to their physico-chemical properties (Figure 3 and Table 1).

As expected, soluble and pure chemicals such as Sodium Benzoate and Diethylene Glycol appeared straightforward to assess. Indeed, for these substances, all three methods employed (OECD 301F, OECD 302B and a modified manometric test for inherent biodegradability) revealed rapid biodegradation, with similar biodegradation kinetics found for the OECD 302B and 301F assays (Figure 3). Sodium Benzoate and Diethylene Glycol passed the 60% threshold and reached, in 28 days, 88–100% of biodegradation for Sodium Benzoate and 78 to 100% of biodegradation for Diethylene Glycol in the employed test methods. In both cases, the 60% pass level was reached within the 10-day window. The 10-day window is defined as beginning when 10% of the degradation is reached and ends after 10 days from this point (but before the 28th day). Detailed information pertaining to biodegradability results are presented in Table 2. Comparison of the three methods (Table 2) shows that the modified manometric test for inherent biodegradability shortens the time needed for Diethylene Glycol to reach the biodegradation pass level compared to the OECD 301F test, while no time gain is observed for Sodium Benzoate because its biodegradation is already very rapid (OECD, 1992a). Our results indicate that these 2 chemicals that biodegrade fast in these tests are consistent with inherent ultimate biodegradability according to OECD 302B and the modified manometric test for inherent biodegradability and as Readily Biodegradable according to the OECD 301F method. These results are in accordance with the literature which define sodium benzoate and diethylene glycol, respectively, as ready and inherently biodegradable (Painter and King, 1985; Lapertot and Pulgarin, 2006; Comber and Holt, 2010).

**Table 2:** Biodegradation parameters of the reference substances tested using the three comparative methods (with n.a. as not applicable; 10 d w. as: 10-day windows; d_60%/70%_ as time to reach the biodegradation pass level).

Chemicals	Sodium Benzoate			Diethylene Glycol		
Methods	BioD (%)	10 d w.	d60%/70%	BioD (%)	10 d w.	d60%/70%
OECD 301F	97% +/−3%	Yes	5	79% +/−3%	Yes	12
OECD 302B	100% +/−0.5%	n.a.	3	101% +/−7%	n.a.	7
The modified manometric test for inherent biodegradability	88% +/−3%	Yes	4	78% +/−1.5%	Yes	6

For the 2 pure soluble compounds, no particular benefits are offered by the modified manometric test for inherent biodegradability compared to the original OECD 302B, which provide comparable outcomes for both methods. In the two methods, the reference substances pass the threshold biodegradation value in less than 14 days, as stipulated for reference substances in the OECD 302B guideline. The range of biodegradation from 80 to 100% is consistent with extensive biodegradation of the substance. Therefore, for this substance category, the main interest in carrying out a modified manometric test for inherent biodegradability over the standard OECD 302B test is the opportunity for automation of the former method.

### 3.2 Comparative assessment of polymer biodegradation using OECD 301F, OECD 302B and the modified manometric test for inherent biodegradability

**Figure 4:**
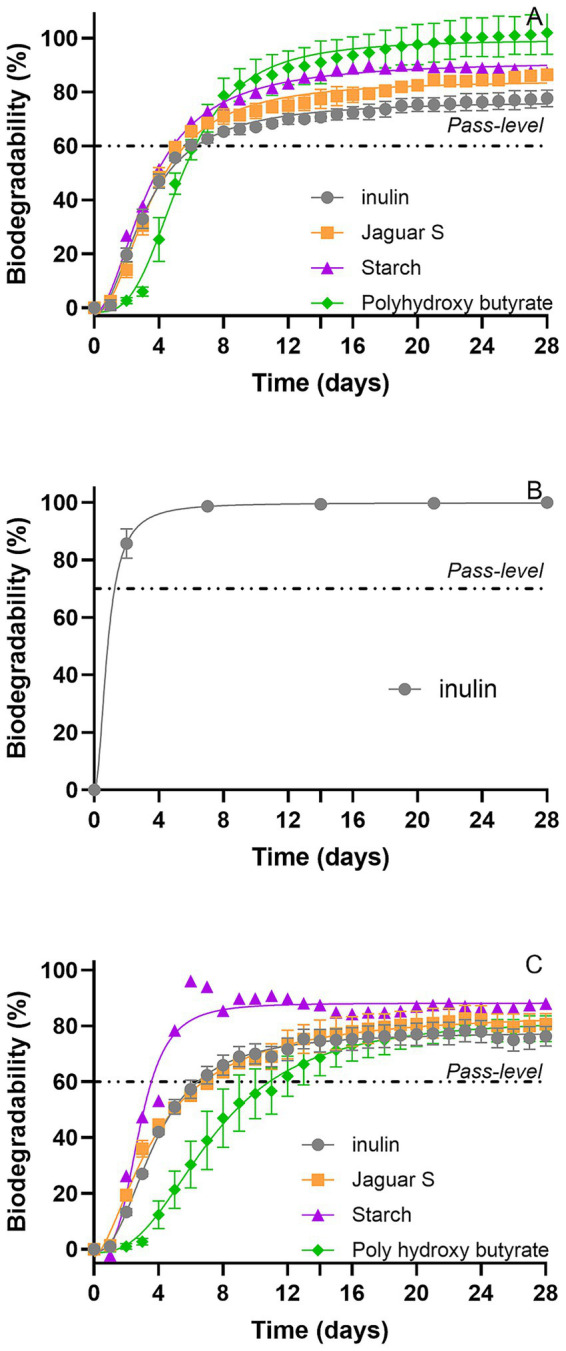
Biodegradability using OECD 301F **(A)**, OECD 302B **(B)**, and the proposed modified manometric test for inherent biodegradability **(C)** with polymeric substances considered as reference. For manometric measurements, biodegradation percentages were calculated relative to measured COD for Inulin, Jaguar S, and Starch, and relative to ThOD for polyhydroxybutyrate.

Applying the 302B test method to characterize the biodegradability of complex chemicals such as polymers and notably insoluble polymers is more challenging. Assessing polymeric substances with reported water solubility in an OECD 302B method may be hampered due to the difficulty to solubilize (especially for high molecular weight, branched or crosslinked polymers), filter or limit the adsorption of these products onto sludge or glassware (Pagga, 1997). Considering the 4 polymers tested, only Inulin, owing to its low molecular weight and high solubility in water appears to be a straightforward material that could be assessed in OECD 302B and passes successfully the centrifugation/filtration steps serving, respectively, to separate the polymer from the inoculum (case of adsorption) and remove free living cells from the sample (as poorly soluble, high-molecular-weight polymers tend to be retained on the filter, thereby preventing proper cell separation). The 4 polymers yielded similar fast and ultimate biodegradation with the two manometric methods OECD 301F and the modified manometric test for inherent biodegradability (Figure 4) with 28-day values in the three test methods ranging from 76 to 100% for Inulin; from 82 to 87% for Jaguar S; from 91.5 to 88% for Starch (Table 3). Among the tested polysaccharides, Inulin behaved as an expected reference material, while Jaguar S and Starch exhibited even faster biodegradation, reaching their pass level within 5–6 days in both manometric methods. Although the 10-day window is not applicable to polymers, all tested polymers met this criterion. Our results indicate that these 3 polymers biodegrade fast in biodegradability screening tests and support their classification as inherently ultimately biodegradable under the applied test conditions according to the modified manometric test for inherent biodegradability and as Readily Biodegradable according to the OECD 301F method. These results are in good agreement with the publicly available data disseminated in the European Chemical Agency web portal.^1^ Indeed, polymers based on natural polysaccharides such as Starch from glucose, Guar Gum from galactose and mannose and Inulin from fructose, are generally considered fully biodegradable and lend themselves well for polymer reference materials in biodegradation assessments. Comparison of the three methods (Table 3) shows that the modified manometric test for inherent biodegradability enables the assessment of polymers that cannot be reliably tested in OECD 302B, while for the polymers that already biodegrade very quickly, the time to reach the pass level is similar in OECD 301F and the modified manometric test for inherent biodegradability, which reflects the biodegradability of these materials.

**Table 3:** Biodegradation parameters of the reference substances tested using the three comparative methods (with: n.a. as not applicable; 10 d w. as: 10-day windows; d_60%/70%_ as time to reach the biodegradation pass level).

Chemicals	Inulin			Jaguar S			Starch			Polyhydroxybutyrate		
Methods	BioD (%)	10 d w.	d_60%/70%_	BioD (%)	10 d w.	d_60%/70%_	BioD (%)	10 d w.	d_60%_	BioD (%)	10 d w.	d_60%/70%_
OECD 301F	76% ± 2%	Yes	6	82% ± 2%	Yes	6	91.5% ± 2%	Yes	5	97% ± 3%	Yes	7
OECD 302B	100 ± 0.5%	*n.a.*	2	*n.a.*	*n.a.*	*n.a.*	*n.a.*	*n.a.*	*n.a.*	*n.a.*	*n.a.*	*n.a.*
The modified manometric test for inherent biodegradability	78 ± 4%	Yes	7	87 ± 4%	Yes	7	88 ± 3%	Yes	5	84 ± 5%	Yes	13

Polyhydroxybutyrate, an insoluble polyester, achieved 97 and 84% mineralization, respectively, for OECD 301F & the modified manometric test for inherent biodegradability (as measured by O2 consumption), indicating a fast and extensive biodegradation (Figures 4A,C and Table 3). Polyhydroxyalkanoates are polyesters naturally produced by certain bacteria as an energy and carbon reserve (Elain et al., 2016). They are well known as biodegradable materials that can be broken down by a variety of microorganisms present in the environment (Deroine et al., 2015; Oliver-Cuenca et al., 2024).

Applying the modified manometric test for inherent biodegradability enabled us to assess the inherent biodegradability of chemicals of complex structure such as polymers for which solubility, adsorption, and agglomeration can complicate their evaluation.

### 3.3 Application of the methodology in the assessment of synthetic polymers

Synthetic polyesters are considered a promising class of biodegradable water-soluble or dispersible materials. After evaluating the modified manometric test for inherent biodegradability on a panel of reference substances representative of different physicochemical situations, we investigated a panel of monomers commonly used in the synthesis of water-soluble polyesters using OECD 301F and OECD 302B. This complementary analysis was performed to support the interpretation of the biodegradation behavior of the synthetic polymer BMT-C*, which was subsequently assessed using OECD 301F, OECD 302B, and the modified manometric test for inherent biodegradability.

**Figure 5:**
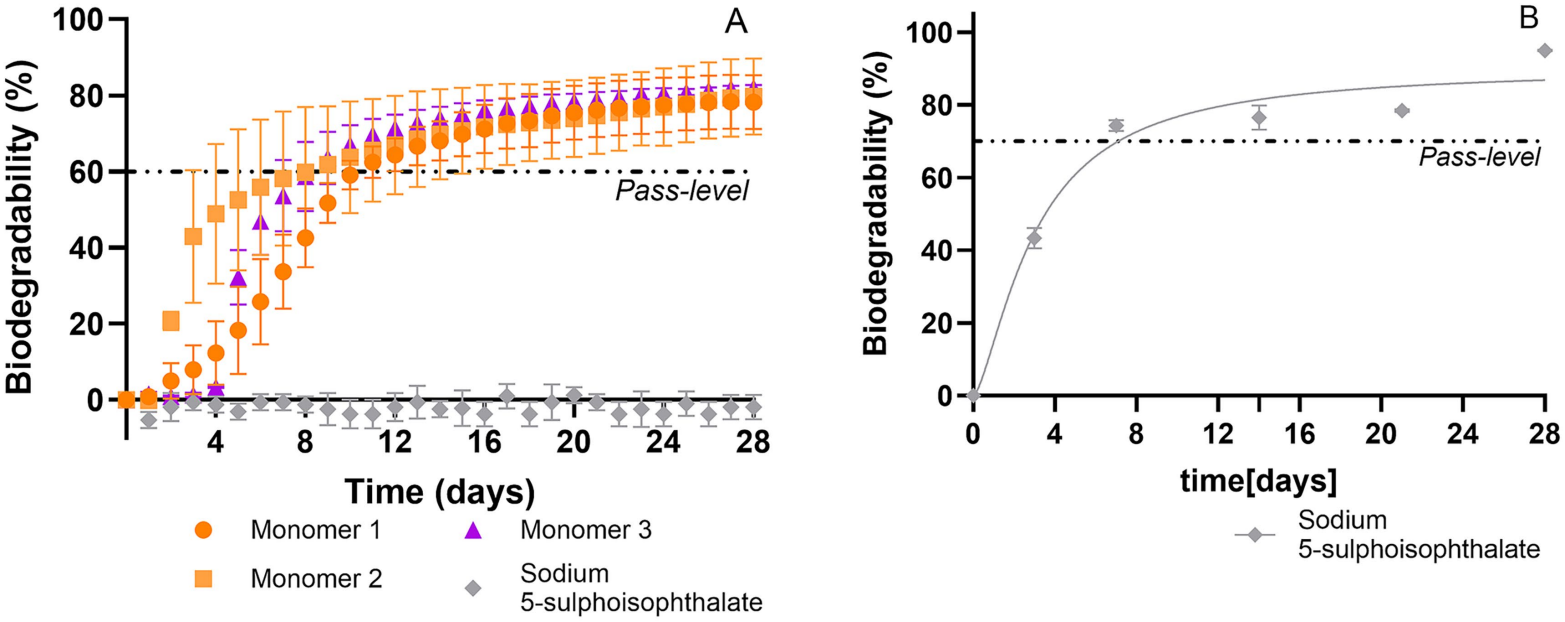
Biodegradability using OECD 301F **(A)** and OECD 302B **(B)** of monomers serving in the preparation of a polymeric substance: BMT-C*. For manometric measurements, biodegradation percentages were calculated relative to ThOD.

A selection of a panel of 4 monomers including notably monomer number 4: Sodium 5-Sulphoisophthalate (SSIA), registered under CAS number: 6362-79-4, were assessed by OECD 301F and OECD 302B for SSIA (Figure 5). Monomers 1, 2 and 3 were concluded as readily biodegradable, reaching the biodegradation threshold of 60% within the 10-day window (Figure 5A). Concerning the SSIA, it appeared as not readily biodegradable, with no mineralization observed under the stringent OECD 301F test condition (Figure 5A), whereas its apparent biodegradation (as measured by dissolved organic carbon) reached a 70% pass level in the OECD 302B within 7 days, indicating an inherent ultimate biodegradability (Figure 5B).

**Figure 6:**
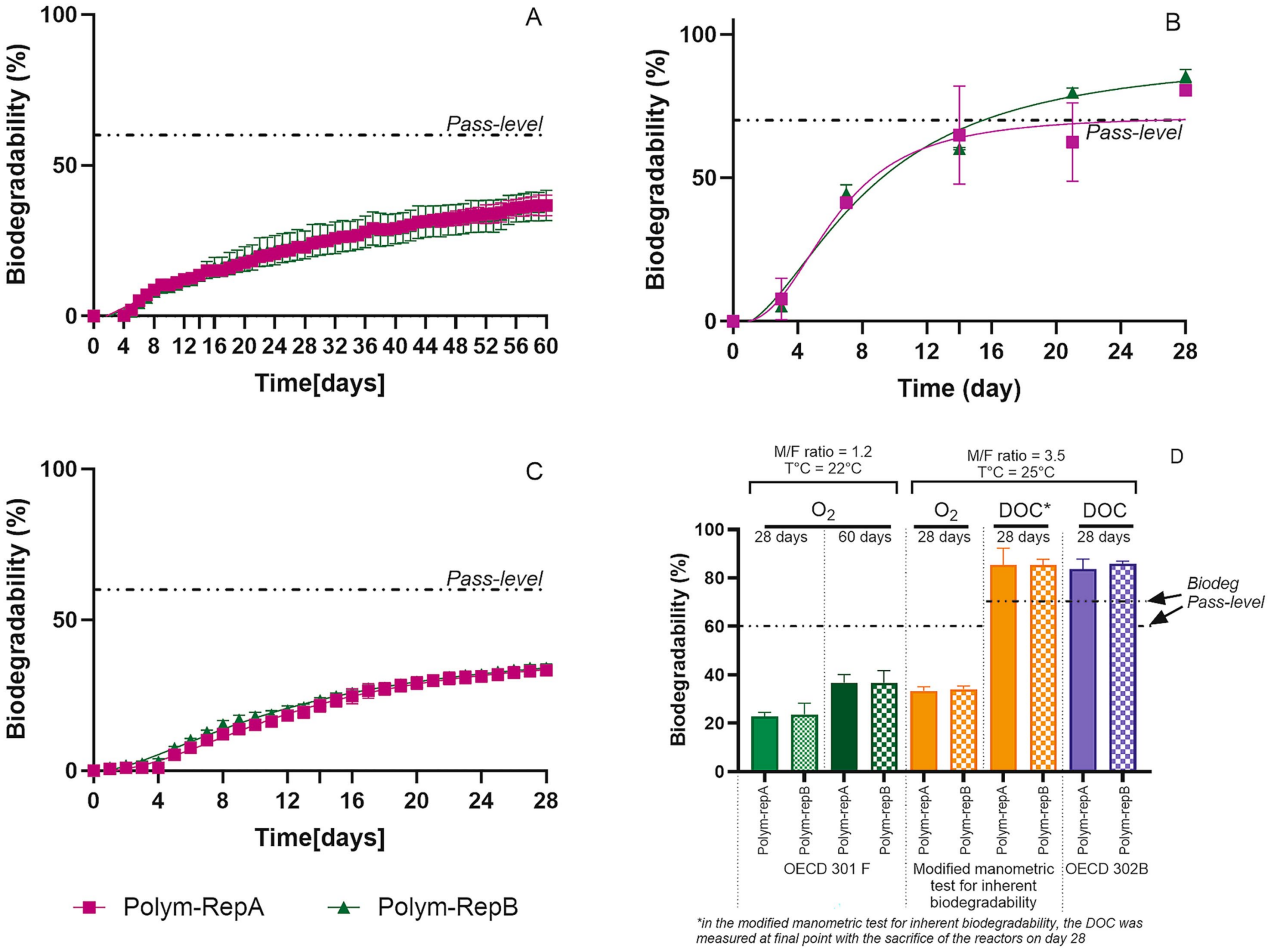
Biodegradability of polymer BMT-C* assessed by **(A)** OECD 301F (manometric O_2_ uptake) (*n* = 3); **(B)** OECD 302B (DOC disappearance) (*n* = 2); **(C)** modified manometric test for inherent biodegradability (manometric O_2_ uptake) (*n* = 2); and **(D)** modified manometric test for inherent biodegradability (DOC disappearance) (*n* = 2). Curves show mean values; shaded ribbons indicate ±SD. Dashed horizontal line marks the 60% or 70% pass level. **(C,D)** came from the same set of bottles which were sacrificed before and after the biodegradation run to allow an accurate comparison between manometric and initial and final DOC-based readouts. Inoculum loading and reactor volume are reported in the Methods section. For manometric measurements, biodegradation percentages were calculated relative to measured COD.

The composition of the polymer BMT-C* consisted of 85% monomers 1 to 3 and 15% SSIA. The biodegradation assessment of BMT-C*, which is composed of 85% readily biodegradable monomers (w/w), was conducted using OECD 301F and included additional analyses to establish its biodegradation pattern (Figure 6). After 28 days, biodegradation reached 23% (± 5%) in the OECD 301F ready biodegradability test. As polymer degradation may be limited by an initial defragmentation step, which can slow the overall biodegradation rate, the test duration was extended to determine whether a degradation plateau could be reached (Figure 6A). According to the revised introduction of the OECD guideline (OECD, 2006), extending the incubation period of ready biodegradability tests up to 60 days may be appropriate when assessing ultimate biodegradation rather than ready biodegradability. However, for polymer BMT-C*, this extended duration was still insufficient to reach the pass level threshold for ultimate biodegradation, with only 37% (± 6%) biodegradation observed for RepA and RepB after 60 days. These results indicate that BMT-C* cannot be considered readily biodegradable.

Applying both OECD 302B and the modified manometric test for inherent biodegradability revealed contrasting results. The OECD 302B test, based on the disappearance of organic carbon, showed apparent polymer mineralization within 15 days, reaching a maximum of 72 and 93% biodegradation after 28 days for Polymer-RepA and RepB, respectively (Figure 6B). Consistent biodegradation results were independently obtained at the Research Center of Shanghai for both OECD 301F and OECD 302B tests, confirming the reproducibility of the data presented in this study (Figures 6A,B; Supplementary Data, Figure A1 in Supplementary material). In contrast, the modified manometric test for inherent biodegradability showed apparent mineralization of 33% & 34 and 84% & 86% after 28 days, respectively, for manometric and organic carbon disappearance measurements (Figures 6C,D). On day 28, the DOC-based readout (Figure 6D) was significantly higher than the manometric readout (Figure 6C), indicating a divergence between oxygen consumption and DOC disappearance data for BMT-C*.

In the case of BMT-C*, the control procedure for polymer adsorption indicated no significant adsorption potential at the study’s start, with over 95% mass recovery after 3 h of pre-incubation (SI appendix section n °1). No initial evidence was found to reject the applicability of the OECD 302B test for BMT-C*. Comparing the modified manometric test for inherent biodegradability results for biodegradation through manometric measurement and organic carbon disappearance suggests that the significant difference between these results may be due to the release of primary biodegradation products with adsorption potential onto biomass or glass.

**Figure 7:**
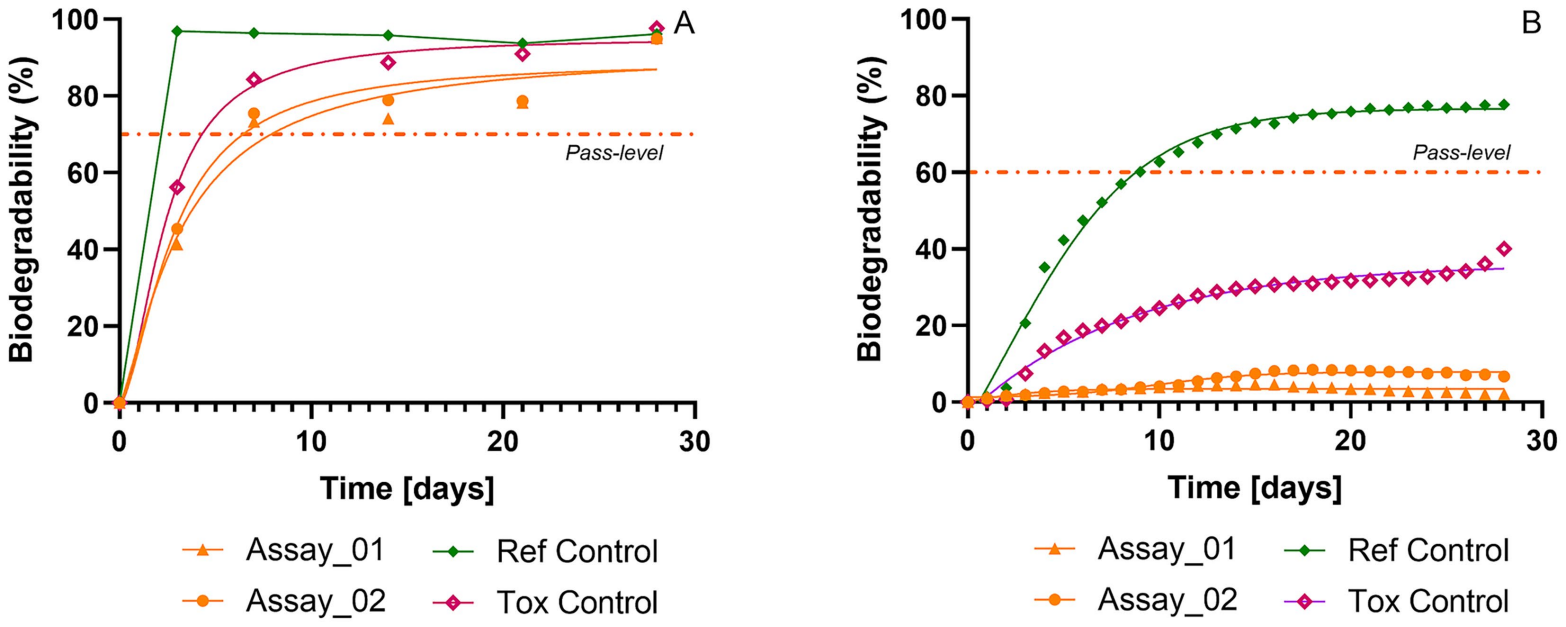
Biodegradability of sodium 5-sulphoisophthalate (SSIA) assessed using OECD 302B **(A)** and the modified manometric test for inherent biodegradability **(B)**. Sodium benzoate was used as the reference substance (green). Test replicates are shown in orange, and the toxicity control (50/50 mg of sodium benzoate and SSIA) is shown in pink. The dashed lines correspond to the respective pass levels for each test. For manometric measurements, biodegradation percentages were calculated relative to ThOD.

Additional analytical support for the non-ultimate biodegradability of polymer BMT-C* was obtained through chromatographic analyses, which indicated the release of degradation products smaller than the parent synthetic polymer, whose initial molar mass was < 15 kDa. In addition, the composition of these degradation products was further investigated by analysing the organic matter recovered in the solid fraction after evaporation of the mineral medium, using NMR. This analysis confirmed the presence of signals attributed to SSIA within the residual polymeric/MES fraction (data not shown). Further analysis of the biodegradability of SSIA was carried out using the modified manometric test for inherent biodegradability. This additional test was performed to evaluate the potential adsorption of degradation products generated during the initial stages of degradation. The OECD 302B method indicated 95% (±1%) disappearance of SSIA at 28 days, with the pass level reached before day 7 (Figure 7A). In contrast, the modified manometric test for inherent biodegradability showed no notable biodegradation over the 28-day incubation period. No significant toxicity was observed in either test (Figure 7B). Taken together, these results suggest that the disappearance of SSIA in the OECD 302B test is unlikely to reflect true biodegradation. It more plausibly results from adsorption during incubation. In the REACH disseminated dossier available through the ECHA webportal (chem.echa.europa.eu), it has been shown that SSIA can significantly adsorb on certain types of soil (although the sorption mechanisms are not understood). In conclusion, the adsorption behavior of the degradation products of the polymer BMT-C* during the biodegradation event may originate from the presence of SSIA in them, although SSIA alone is unlikely to fully explain the divergence observed between the two methods.

## 4 Conclusion

The study aimed to evaluate the effectiveness of the modified manometric test for inherent biodegradability as a complementary approach for assessing the biodegradability of various substances, including polymers. The results indicate the relevance of this approach, particularly in situations where traditional methods may face important limitations.

The modified manometric test for inherent biodegradability was compared with standard biodegradability tests, including OECD 301F and OECD 302B. Soluble and pure chemicals such as Sodium Benzoate and Diethylene Glycol were effectively assessed using all three methods, achieving biodegradation levels between 78 and 100% within 28 days. These results indicate that the modified manometric test for inherent biodegradability provides outcomes comparable to traditional methods, with the added benefit of automation.

The modified manometric test for inherent biodegradability enabled the assessment of polymers such as Inulin, Jaguar S, Starch, and Polyhydroxybutyrate. These polymers achieved biodegradation levels ranging from 76 to 100%, supporting their overall inherent biodegradability. Its application to synthetic polymers, such as polymer BMT-C*, highlighted the main advantage of the method by mitigating the risks associated with the inadequacy of the OECD 302B method for testing non-soluble or adsorptive compounds that can be released over the incubation period. Overall, the study supports the modified manometric test for inherent biodegradability as a useful complementary approach for biodegradability assessment, particularly for polymers and other substances for which DOC-based methods may be difficult to interpret.

Future research should focus on refining the method to broaden its applicability to chemistries covering other pure chemicals and polymers including those with challenging physicochemical properties. It would also be pertinent to evaluate the potential of implementing bioavailability improvement methods proposed in the OECD 301F method. Additionally, further exploration of the method’s automation potential could streamline biodegradability assessments, making them more efficient and accessible. In this context, the modified manometric test for inherent biodegradability appears as an interesting complementary approach alongside OECD 302B and 302C.

In conclusion, modified manometric test for inherent biodegradability represents a promising incremental advancement in biodegradability testing for complex and poorly soluble substances. However, the present study remains limited by the number of substances tested and by the use of bulk readouts that do not resolve the fate of individual constituents. For complex materials such as polymers, oxygen uptake alone should also not be interpreted as definitive proof of complete mineralization without complementary measurements. Finally, multi-site ring-testing and alignment with OECD acceptance criteria, together with evaluation of a broader range of substances across varied conditions, repeated experiments, and different inocula, will be essential steps toward standardization and regulatory acceptance, and to better establish the reproducibility, applicability, and overall robustness of the method.

## Data Availability

The original contributions presented in the study are included in the article/Supplementary material, further inquiries can be directed to the corresponding author.

## Footnotes

1
https://chem.echa.europa.eu/
