# Supplementary material for: A novel manometric test to assess inherent biodegradability of complex and non-soluble chemicals
Source: Front Microbiol. 2026 Apr 24;17:1822141. doi: 10.3389/fmicb.2026.1822141 (PMC13153034; doi:10.3389/fmicb.2026.1822141)

Supplementary Material

# Supplementary Data –modified manometric test for inherent biodegradability -General Protocol

## Method:

### Preparation of the test

# General Implementation:

# Minimal setup to plan the assessment of 1 substance

# Run 2 inoculum blanks

# Run 2 test flasks per substance studied

# Run a toxicity flask per test substance studied

# Run a reaction control flask to validate the activity of the inoculum, using a reference substance

# Optionally run one or more abiotic flasks for the test substance studied

# Add the calculated volume of test medium for each flask

# Add the pre-prepared test substance according to concentrations between 50 to 250 mg/L of DThO

# Add between 100 to 400 mg/L of SS using the prepared inoculum

# Add NaOH pellets for CO_2_ trapping

# Seal the flasks tightly and install the Oxitop™ devices

### Test: Launch and Monitor

# Place the flasks in the dark in an incubator at 20-25°C

# Use the Bluetooth Multi 3620 system to record oxygen consumption

# Monitor the test by collecting raw data using the Multi 3620 control box. These results are then transferred to a data sheet for subsequent analysis

### Post-analysis: Validation and Termination of the assay

# The test duration is theoretically 28 days but can be more or less extended

# Degradation of the reference substance must be greater than 60% in 14 days

# Oxygen consumption in the controls is normally between 100 and 150 mg O2.L ^-1^ and should not exceed 300 mgO_2_/L

# Ensure that variability between replicates is less than or equal to 20%

# The toxicity control must show a biodegradation threshold greater than or equal to 25% on the 14th day of incubation

# The pH measured at the end of the test should be between 6-8.5

## Materials & Reagents:

### Materials

# Necessary Equipment:

# Thermostatic incubator (20-25°C)

# Refrigerated centrifuge and its bottles

# Ovens at 105°C

# Thermobalance

# Ultra-Turrax

# pH meter

# Bottle or Jars with Oxitop™ heads and remote control (Xylem, FR) (total volume of 510mL or 2,580 mL with magnetic stirrers and pressure caps)

# Bottle used in the assay can be the standard 510mL brown flask WTW PF600: WW209100,

# In the case of the use of a jar of 2,580 mL, the reference is OxiTop®-IDS B6M-2.5

# NaOH pellets for CO2 trapping

# Automatic pipettes

# Weighing balance

# Standard glassware

# Spectrophotometer

### Reagents

# Various reagents:

# Sodium hydroxide NaOH: 1 - 2N

# Buffer solutions for pH 4, 7, 10

# Hach reagents for COD measurement (range 150-1000 mg. L ^-1^)

# NaOH pellets

# HCL 1N (dilution of a concentrated solution)

# Mineral Medium Preparation:

# Mineral medium prepared from solutions a, b, c, and d as described in the OECD guideline

# Solution a:

# Anhydrous potassium dihydrogen phosphate KH_2_PO_4_....................... 8.50 g

# Anhydrous potassium monohydrogen phosphate K_2_HPO_4_ ............. 21.75 g

# Sodium monohydrogen phosphate dihydrate Na_2_HPO_4_, 2H_2_O........ 33.40 g

# Ammonium chloride NH_4_Cl....................................................................0.50 g

# Demineralized water .......................................................             qsp 1,000 mL

# Solution b:

# Magnesium sulfate heptahydrate MgSO_4_, 7H_2_O................................ 22.5 g

# Demineralized water ...................................................................qsp 1,000 mL

# Solution c:

# Calcium chloride dihydrate CaCl_2_, 2H_2_O............................................. 36.4 g

# Demineralized water....................................................................qsp 1,000 mL

# Solution d:

# Ferric chloride hexahydrate FeCl_3_, 6H2O ............................................ 0.25 g

# Demineralized water ...................................................................qsp 1,000 mL

# Note: Solutions a, b, c, and d can be autoclaved and stored at 4°C for easier preservation, or a drop of HCl can be added to stabilize the solution and allow its preservation. Solutions a, b, c, and d must be renewed at least annually. A complementary NH4Cl solution prepared on the day or stored sterile at 4°C can be added to increase the elemental nitrogen supply if needed. The ideal incubation medium ratio is around 10/1 (C/N).

# The test system will be prepared as follows: For 1 liter:

# 10 mL of solution a

# 1 mL of each of solutions b, c, and d

# Demineralized water qsp 1,000 mL

# *The pH of the prepared medium must be checked and adjusted if necessary to 7.2. The pH is checked on the day of use with the pH meter.*

## Extemporaneous preparations:

# Preparation of the Test item/ Substance

# Calculate the Theoretical Oxygen Demand (DThO) from the empirical formula

# Know the solubility of the product and if not, perform a COD analysis, if insoluble, perform a direct addition

# Preparation of the Inoculum

# Use activated sludge from a station and determine the Suspended Solids (SS) content of the biomass by centrifuging a known volume. The pellet obtained is dried using an oven set at 105°C for ideally 24 hours until a constant weight is obtained. Weigh and calculate the concentration of Suspended Solids (SS) expressed in Dry Mass (DM) (mg. L ^-1^).

# Wash the inoculum according to the protocol, adjusting the final concentration to 2.5 g/L of suspended solids (SS). For this:

# Place 150g of inoculum per jar (3 jars)

# Centrifuge for 15 min, 6,400 RCF and remove the supernatant

# Resuspend in the mineral medium

# Pass through Ultra-Turrax for 10 seconds

# Adjust the inoculum to a final volume of 100g per jar

# Repeat these steps 3 times

# Then combine the 3 jars into 2 jars

# Centrifuge

# Remove the supernatant

# Adjust the inoculum to the desired final concentration of 2.5 g/L

# Measure the SS immediately:

# Centrifuge 2 flasks of sludge (100g) and remove the supernatant

# Recover all the pellet and place in an aluminum dish (previously weighed empty)

# Incubate overnight in the oven at 105°C degrees

# The next day, record the weight and mathematically determine the SS

# Supplementary Table. A1.

An overview of the different testing conditions applied using the Modified manometric test for inherent Biodegradability in this study. Data were evaluated in terms of signal interpretation, to assess the robustness of the experimental setup (Supplementary Table A1). For this purpose, a signal-to-noise ratio (S/N) was calculated (Eq: A1), where noise is defined as the variability of blank respiration. Here, µ_x_​ represented the measured BOD in the test assay, µ_basis_​ is the mean blank respiration, and σ_basis_​ is the standard deviation of the blank.

Despite relatively high baseline respiration under some conditions, the inoculum-to-food ratio and the filling volumes of the test vessels resulted in low variability in blank measurements, yielding high absolute S/N values. This demonstrates that the signal remains well resolved and that the method is reliable (Table A1). The baseline signal corresponds to the endogenous respiration of the blank inoculum (expressed as O₂ consumption), whereas the measured BOD reflects the oxygen demand associated with microbial degradation of the test substance.

Eq A1:

$$\frac{Signal}{Noise}=\frac{\left( \mu_{x}-\mu_{basis} \right)}{\sigma_{basis}}$$

Supplementary data Table A1 Conditions of test done with the Modified manometric test for inherent Biodegradability during this study. Overall, the experimental setup exhibited high signal-to-noise (S/N) ratios, indicating that the biodegradation signal was well resolved and only minimally affected by background noise under the tested conditions

| Parameters / Chemical | Sodium benzoate | DEG | starch | DEG | Jaguar S | Inulin | P3HB |
| --- | --- | --- | --- | --- | --- | --- | --- |
| Biomass (mg MLSS L^-1^) | 400 | 400 | 71 | 400 | 71 | 71 | 71 |
| Filling Volume (mL) | 500 | 500 | 500 | 500 | 1000 | 1000 | 500 |
| Total Volume (mL) | 2,580 | 2,580 | 2,580 | 2,580 | 2,580 | 2,580 | 2,580 |
| Initial O₂ available (t₀) (mg O_2_) | ~ 576 | ~ 576 | ~ 576 | ~ 576 | ~ 438 | ~ 438 | ~ 438 |
| Expected blank respiration (mg O_2_) | 200 ~ 260 | 200 ~ 260 | 200 ~ 260 | 200 ~ 260 | 71 - 91 | 71 - 91 | 71 - 91 |
| Measured blank respiration (mgO_2_) | 215 (±5) | 215 (±5) | 215 (±5) | 215 (±5) | 36 | 36 | 36 |
| Theoretical O₂ demand for 100% biodegradation (COD / ThOD) (mg O_2_) | 143 | 91 | 50 | 91 | 139 | 134 | 151 |
| Measured BOD  (mg O_2_) | 121 (± 5) | 80 (± 5) | 44 (±0) | 80 (±5) | 111 (± 7) | 103 (± 5) | 120 (± 5) |
| Signal-to-noise ratio (S/N) | ~ 19 | ~ 27 | ~ 34 | ~ 27 | ~ 15 | ~ 13 | ~ 17 |

# Supplementary Fig. A1.

**Supplementary Fig. A1.**

Biodegradation results of the BMT-C* polymer were obtained both in France and in China. Comparable results were obtained at the two sites, using independent sludges. In the French laboratory, the OECD 301F test gave 23% (± 5%) and 37% (± 6%) after 28 and 60 days, respectively (Fig. A1.A). In the Chinese laboratory, the OECD 301F test gave 32% (± 6%) and 45% (± 9%) after 28 and 60 days, respectively (Fig. A1.C). For the OECD 302B test, results showed ultimate biodegradability, after 28 days, with biodegradation percentages of 82.5% (± 10%) and 95.6% (± 4%), respectively in the French (Fig. A1.B) and in the Chinese laboratory (Fig. A1.D).

Fig. A1 Biodegradability of polymer BMT-C* assessed in Lyon-France by (A) OECD 301F (manometric O₂ uptake) (n = 3) and (B) OECD 302B (DOC disappearance) (n = 2); and assessed in Shanghaï-China by (C) OECD 301F (manometric O₂ uptake) (n = 2) and (B) OECD 302B (DOC disappearance) (n = 2). Curves show mean values; shaded ribbons indicate ±SD. Dashed horizontal line marks the 60% or 70% pass level.

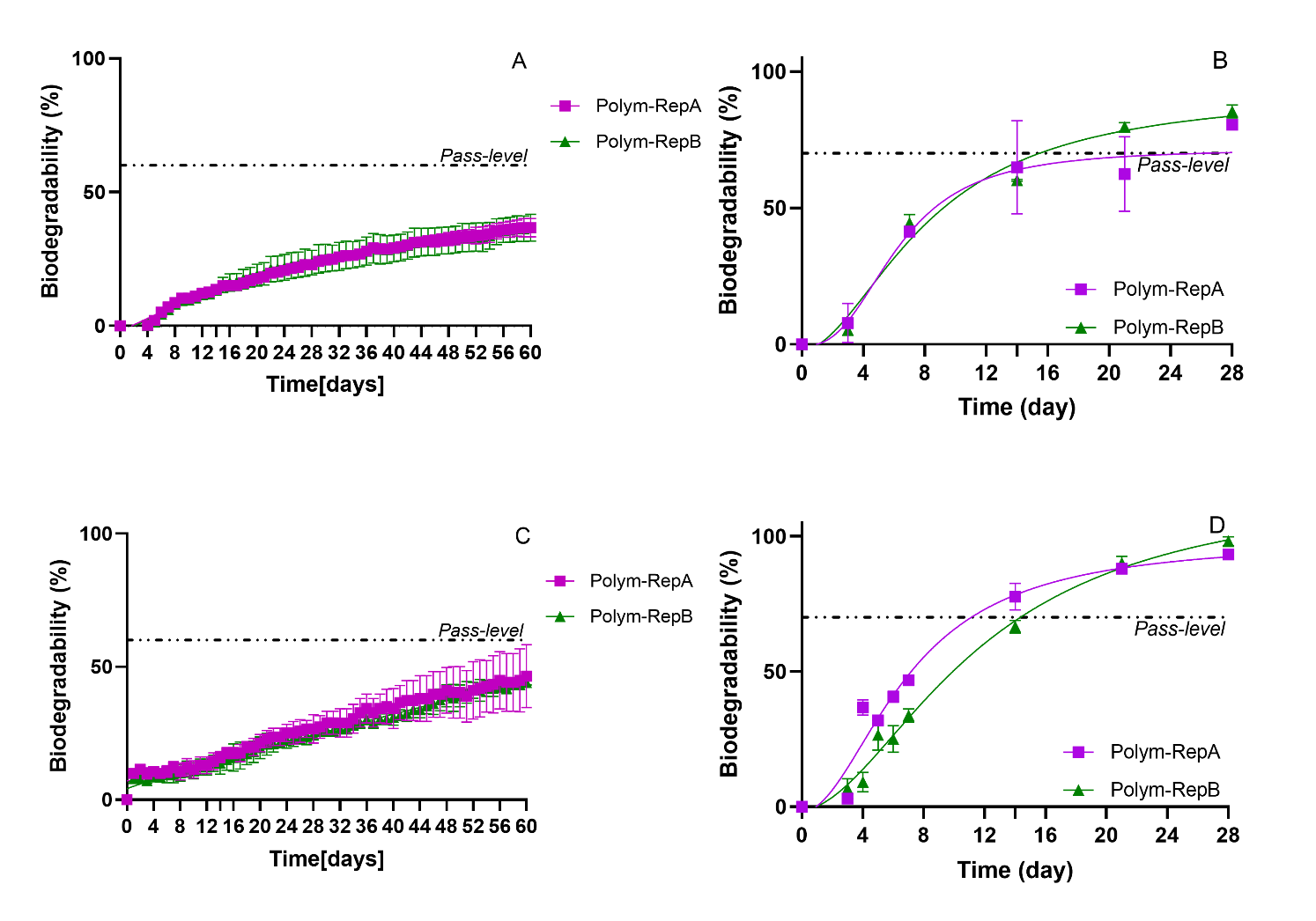

Supplement: Supplementary file 1 [file Table_1.docx]
